# Rational Design of High-Performance Hemithioindigo-Based Photoswitchable AIE Photosensitizer and Enabling Reversible Control Singlet Oxygen Generation

**DOI:** 10.3390/bios13030324

**Published:** 2023-02-27

**Authors:** Junjie Wang, Jianshuang Wei, Yuehong Leng, Yanfeng Dai, Changqiang Xie, Zhihong Zhang, Mingqiang Zhu, Xingzhou Peng

**Affiliations:** 1 Britton Chance Center and MoE Key Laboratory for Biomedical Photonics, Wuhan National Laboratory for Optoelectronics, Huazhong University of Science and Technology, Wuhan 430074, China; 2 Key Laboratory of Biomedical Engineering of Hainan Province, School of Biomedical Engineering, Hainan University, Haikou 570228, China; 3 Wuhan National Laboratory for Optoelectronics, School of Optical and Electronic Information, Huazhong University of Science and Technology, Wuhan 430074, China

**Keywords:** photoswitchable, aggregation-induced emission, photosensitizer, hemithioindigo

## Abstract

A photosensitizer furnishing with reversible control singlet oxygen generation (^1^O_2_) is highly desirable for precise photodynamic therapy (PDT), lessening non-specific harm to healthy tissues. Here, a novel photoswitchable aggregation-induced emission (AIE) photosensitizer based on a triarylamine (TPA)-modified hemithioindigo (HTI), 6Br-HTI-TPA-OMe, was rationally designed. The triarylamine AIE photosensitizing moiety and HTI switch unit were covalently linked in one molecule, permitting reversible regulation of ^1^O_2_ production. The photophysical evaluations revealed that 6Br-HTI-TPA-OMe possessed excellent AIE properties and Z/E photoswitch performance in different solvents. Additionally, the amphiphilic phospholipid-fabricated nanoparticles (NPs) also exhibited photochromic behavior in water. The Z-NPs initiated the generation of ^1^O_2_ upon 520 nm light-emitting diode (LED) irradiation, but after switching to E-NPs, the generation of ^1^O_2_ was inhibited by the competitive energy transfer, suggesting that reversible Z/E isomerization could photocontrol ^1^O_2_ generation. The in vitro anti-tumor experiment verified that the 6Br-HTI-TPA-OMe can act as a photoswitchable AIE photosensitizer. This is the first report on the photoswitchable AIE photosensitizer of HTI-based molecules, to the best of our knowledge.

## 1. Introduction

Photodynamic therapy (PDT) is a prospective therapeutic modality for anti-cancer strategies due to its noninvasive nature and local treatment benefits. Generally, PDT causes effective cytotoxicity by converting oxygen to various reactive oxygen species (ROS) by triggering photosensitizer (PS) with light irradiation [1,2]. However, the unavoidable toxicity of traditional PS in the “always-on” mode has been a sticky obstacle in PDT, which causes serious side effects to health tissues [3,4]. Thus, developing the controllable PSs system is highly desirable for precise treatment, which can be activated by pathological stimulations such as pH, hypoxia, or enzymes. However, these regulation processes typically suffer from the inaccurate control of ^1^O_2_ production due to the irreversible means [5].

Recently, a system of light-activatable photosensitizer based on a photoswitch molecule (e.g., azobenzene, spiropyran, diarylethene) has been able to reversibly control the generation of ^1^O_2_ by utilizing the light with noninvasive and spatiotemporal-controlling properties [5,6,7]. For instance, diarylethene (DAE) switches have been developed in conjunction with PS in one molecular structure for reversibly controlling the ^1^O_2_ generation. In general, when DAE was in the open form, PS was triggered; after DAE switched to the closed form, the PS’s photosensitization was blocked. This can be explained by tuning the energy-dissipated pathway of PS with the help of DAE switch unit [8]. However, in a hydrophilic and physiological environment, the majority of them are prone to fluorescence quench and insufficient ^1^O_2_ generation as a result of the aggregation-caused quenching (ACQ) phenomenon, which severely hinders their imaging and PDT therapeutic application. Thus, AIE-based smart materials have been used to address these issues. For example, a fluorescent fluoride chemosensor (SP-OSi/SP-O) with AIE properties was successfully used in microfluidic paper-based analytical devices (μPAD) to detect fluoride, showing a 10-fold improvement in sensitivity [9]. It has recently been successful to develop a photoswitchable PS system (TPE-2Py-DTE) with AIE properties, exhibiting bright emission and reversible control of ^1^O_2_ production for anti-bacteria application [10]. However, UV light (420 nm) was used to stimulate the generation of ^1^O_2_, which is restricted by the penetration of light. Therefore, it still remains a severe challenge to expand the toolkit of longer wavelength (e.g., NIR) responsive PS systems with AIE capability.

Hemithioindigos (HTIs) are asymmetrical compounds comprising a central photoisomerizable C=C olefinic bond that connects a half-thioindigo moiety to half-stilbene moiety [11]. This core olefinic bond can be isomerized between the Z and E configurations under visible light. The barrier for thermal isomerization from E form to Z form (>27 kcal/mol) is high, making them an intrinsically very bistable switching system, which benefits to accumulate the desired isomer in a large amount by adjusting light. Compared to the sophisticated synthetic route of DTE, the synthetic route of HTI derivatives is straightforward [12,13]. Due to these advantages, HTI switches have been utilized in the field of conformational control peptides, such as light-switching of membranes, gramicidin channels, enzymes, and molecular rotors [12,14,15,16,17]. However, the anti-tumor potential of visible light-activated HTI-based molecules has been rarely investigated.

**Scheme 1:**
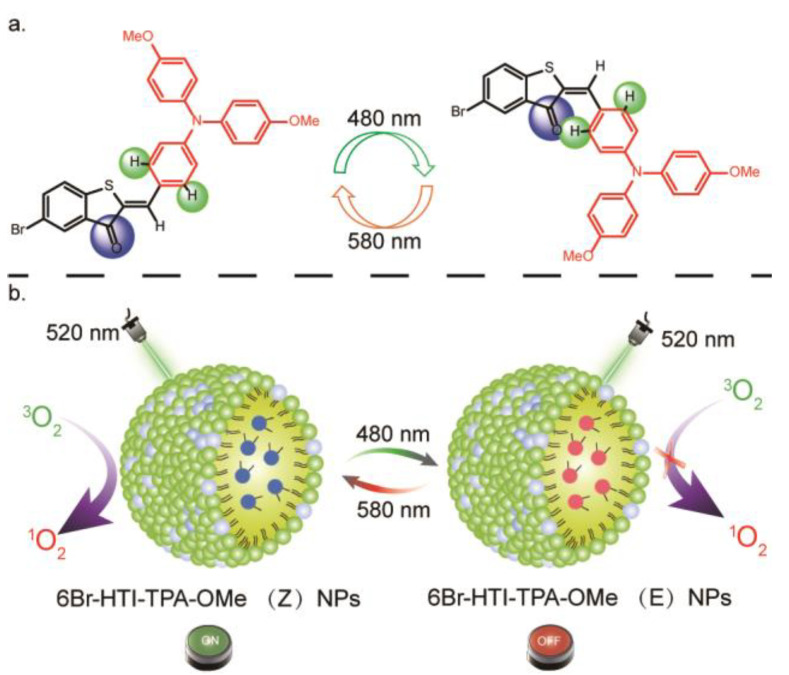
The graphic illustration of a photoswitchable AIE photo-sensitizer for PDT. (**a**) The isomerization of small molecule 6Br-HTI-TPA-OMe; (**b**) schematic illustration of the controllable ^1^O_2_ generation in Z-NPs and E-NPs.

In this work, a rationally designed triarylamine modified HTI photoswitchable AIE PS (6Br-HTI-TPA-OMe) has been developed (Scheme 1). The triarylamine (TPA) fragment and thio-moiety were covalently linked through an olefinic bond. Moreover, the TPA part is an ideal building block for constructing various AIEgens, which have a propeller-like molecular structure and a strong electron-donating property. The bromine was anticipated to facilitate the intersystem crossing (ISC) process, owing to the heavy-atom effect. The electron-donating OMe groups in the TPA and the bromine in the thio-part should stimulate intramolecular energy transfer (ICT) to improve photosensitizing abilities and to encourage photochromism by reducing the HOMO-LUMO energy gap. Owing to the enhanced ICT, the reversible control of ^1^O_2_ generation was feasible. Thus, we speculate that 6Br-HTI-TPA-OMe enables reversible control ^1^O_2_ generation because of the energy discrepancies between the triplet state of AIE PS and the start state of Z and E isomers, respectively. Further studies revealed how 6Br-HTI-TPA-OMe reversibly regulates ^1^O_2_ production by controlling the Z and E forms when exposed to different light wavelengths. The excellent photosensitive performance on the 4T1 cells in vitro verified that a favorable light-activatable AIE PS has been successfully developed.

## 2. Materials and Methods

Materials: If not specially mentioned, the starting materials and solvents were purchased from Sinopharm Chemical Reagent Co. Ltd. (Ningbo, China)and used as received without further purification. 1,2-dimyristoyl-sn-glycero-3-phosphocholine (DMPC), 1,2-distearoyl-sn-glycero-3-phosphoethanolamine-N-propyl ethylene glycol-2000 (DSPE-PEG_2000_) were obtained from Avanti Polar Lipids Inc. (Alabama, USA). Copper(I) chloride (CuCl) was purified by re-precipitation from the concentrated HCl.

### 2.1. Synthesis of 6Br-HTI-TPA-OMe

A mixture of 4-/6-bromobenzo[*b*]thiophen-3(2*H*)-one **5** (160.0 mg, 0.7 mmol, 1.4 equiv.) was dissolved in 10 mL of benzene at room temperature. Then, *N*,*N*′-bis(4-methoxyphenyl)aminobenzaldehyde 2 (170.0 mg, 0.5 mmol, 1.0 equiv.) and one drop of piperidine were added. The reaction mixture was refluxed for 3 h. The solution was concentrated, and the residue was purified by chromatography with pentane/dichloromethane (1/2–1/1, *v*/*v*) to yield 6Br-HTI-TPA-OMe (98.7 mg, 36.3%); ^1^H NMR (500 MHz, CDCl_3_) *δ* = 7.89 (s, 1H), 7.75 (d, ^3^*J* = 8.2 Hz, 1H), 7.64 (d, ^4^*J* = 1.5 Hz, 1H), 7.48 (d, ^3^*J* = 8.9 Hz, 2H), 7.38 (dd, *J* = 8.2, 1.6 Hz, 1H), 7.13–7.10 (m, 4H), 6.91- 6.85 (m, 6H), 3.80 (s, 6H); ^13^C NMR (126 MHz, CDCl_3_) *δ* = 187.43, 157.33, 151.21, 147.60, 139.32, 135.35, 133.07, 130.30, 129.79, 129.04, 128.01, 127.88, 126.81, 125.69, 124.98, 118.31, 115.23, 55.74; HRMS(APCI): *m/z* 544.0575 (97.3%, [*M*(^79^Br)+H]^+^, C_29_H_23_BrNO_3_S^+^, calcd. 544.0577), 546.0554 (100.0%, [*M*(^81^Br)+H]^+^, C_29_H_25_BrNO_3_S^+^, calcd. 546.0556).

### 2.2. Preparation of 6Br-HTI-TPA-OMe NPs

The 6Br-HTI-TPA-OMe NPs were prepared by thin film hydration and then extrusion method. In brief, DMPC, DSPE-PEG_2000_, and 6Br-HTI-TPA-OMe with a molar ratio of 60:2:0.7 were dissolved in chloroform and then dried under nitrogen to form a uniform film. The mixture was then hydrated with 1 mL PBS and sonicated for 1 h at 52 °C. After 1 h, the monolayer liposomes were obtained by an Avanti polar Lipids extruder with pores of 200 nm. Finally, 6Br-HTI-TPA-OMe NPs were obtained.

## 3. Results and Discussion

The photoswitchable AIE photosensitizer 6Br-HTI-TPA-OMe was synthesized as shown in Scheme S1, Supplementary Materials. Firstly, the *N,N’*-(diphenylamino)benzaldehyde **2** was prepared according to the literature [18,19]. Then, a mixture of 4-/6-bromobenzo[b]thiophen-3(2H)-one **5** was formed by Friedel–Crafts acylation. The targeted 6Br-HTI-TPA-OMe was obtained by Knoevenagel condensation of compounds **2** and **5** in a yield of 36.3%. The chemical structure was identified by NMR spectroscopy and high-resolution mass spectrometry (Figures S1–S7).

### 3.1. AIE Properties and Photochromism of 6Br-HTI-TPA-OMe in Organic Solutions

**Figure 1:**
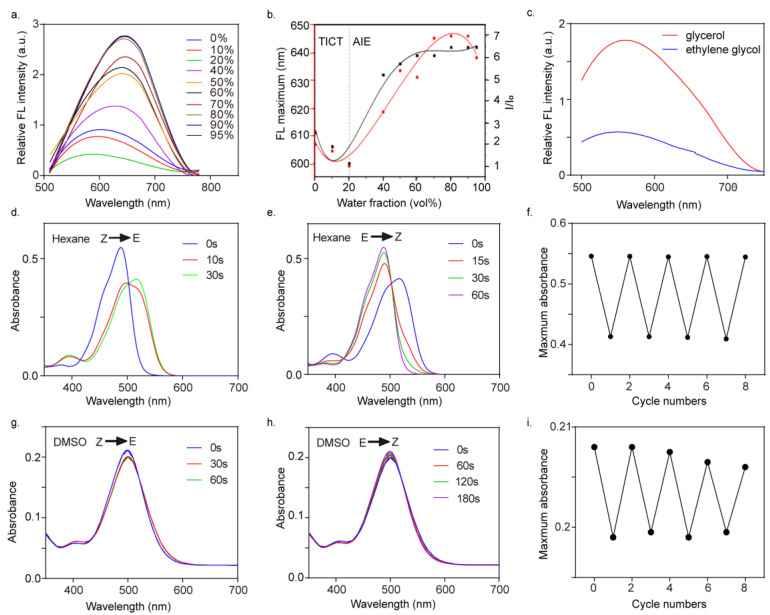
AIE properties and photochromism of 6Br-HTI-TPA-OMe in organic solutions. (**a**) The fluorescence spectra of 6Br-HTI-TPA-OMe in DMSO/H_2_O mixtures with different water fractions (*v/v*) (50 μM, λ_ex_ = 495 nm); (**b**) plot of maximum fluorescence (red) and relative fluorescence intensity (I/I_0_, black) versus the composition of the DMSO/H_2_O mixtures, where I_0_ was the fluorescence intensity at 20% *f*_w_; (**c**) the fluorescence spectra of 6Br-HTI-TPA-OMe in glycerol and ethylene glycol (10 μM, λ_ex_ = 450 nm); the absorption spectra changes (**d**,**e**) and fatigue resistance (**f**) of 6Br-HTI-TPA-OMe in hexane (10 μM) upon switching LED light either with 480 nm or 580 nm; the absorption spectra changes (**g**,**h**) and fatigue resistance (**i**) of 6Br-HTI-TPA-OMe in DMSO (10 μM) upon alternating irradiation light either with 480 nm or 580 nm.

First, we examined the AIE characteristics of 6Br-HTI-TPA-OMe. As depicted in Figure 1a, the maximum emission peak at 611 nm was observed for 6Br-HTI-TPA-OMe in the DMSO solution. The fluorescence intensity was reduced by 2.0 times and a blue-shifted emission from 611 to 600 nm was observed when the water fractions (*f*_w_) in the DMSO/water grew from 0 to 20%. This behavior can be attributed to the twist intramolecular charge transfer (TICT) effect [20,21]. Due to the formation of the aggregates [22], the further addition of water from *f*_w_ = 20% to 95% resulted in a red-shifted emission to 642 nm and an increase in fluorescence intensity of seven times (red line) and relative fluorescence intensity (I/I_0_, black line) (Figure 1b). The change in the absorption and fluorescence spectroscopy with different solvent polarities of 6Br-HTI-TPA-OMe were investigated. The 6Br-HTI-TPA-OMe displayed dual-emissive bands, a large Stokes shift, and fluorescence quenching in more polar solvents (Figure S8 and Table S1), indicating the formation of a twist intramolecular charge transfer (TICT) state in polar solvent. Next, the emission of 6Br-HTI-TPA-OMe in different viscosity solvents was explored. A three-fold increase in emission intensity was performed in glycerol (*η* = 945 mPa·S) compared to ethylene glycol (*η* = 13.5 mPa·S), demonstrating that the restriction of intramolecular motion is the main reason for the AIE phenomenon [23]. The fluorescence quantum yield of Z-NPs was 3.7%, which is higher than that in polar solvents (DCM: Φ*_f_* = 0.06 and MeCN: Φ*_f_* = 0.01), resulting from the AIE aggregate in the core of the nanostructure.

The photochromic behaviors of 6Br-HTI-TPA-OMe were explored in different solvents. As illustrated in Figure 1d, the Z isomer showed an absorption peak at 480 nm in hexane. After 30 s of irradiation with 480 nm visible light, a new absorption maximum at 540 nm appeared, suggesting the formation of E isomers. Furthermore, a unique isosbestic point at 520 nm was detected, suggesting that two isomers underwent a photochromic transition. The E-to-Z reverse reaction was triggered by exposure to orange-red light (580 nm, 60 s), as shown in Figure 1e. Additionally, such isomerization could be carried out in five cycles without discernible attenuation, demonstrating that 6Br-HTI-TPA-OMe has strong reversibility (Figure 1f). Next, photochromic behavior in the polar solvent (methyl sulfoxide, DMSO) was evaluated and triggered with the same light (Figure 1g–i). Due to the solvent-dependent photochromic performance, very weak photochromic behaviors in DMSO were detected.

### 3.2. The Photochromic Behavior of 6Br-HTI-TPA-OMe NPs

**Figure 2:**
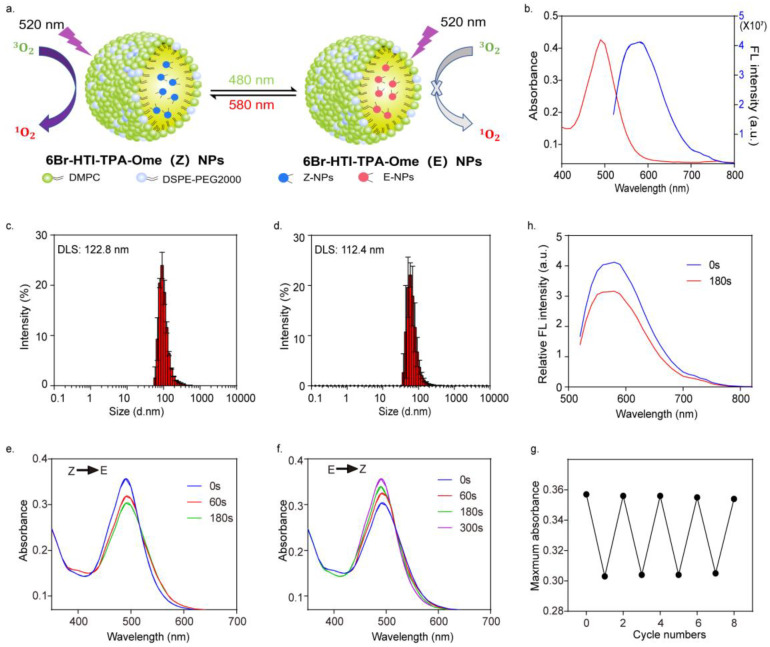
The photochromic behavior of 6Br-HTI-TPA-OMe NPs. (**a**) Schematic diagram of controllable ^1^O_2_ generation in Z-NPs and E-NPs; (**b**) the absorption (red line) and fluorescence (blue line) spectra of Z-NPs (10 μM; Ex: 450 nm); dynamic light scattering (DLS) profiles of Z-NPs (**c**) and E-NPs (**d**); the absorption spectra changes of NP (25 μM) upon alternating irradiation with 480 nm (**e**) and orange-red light at 580 nm (**f**); (**g**) the fatigue resistance of NPs for five cycles in water; (**h**) the fluorescence spectra change of NPs upon irradiation with 480 nm.

To improve the hydrophobic compound with water solubility and in vivo biocompatibility, the 6Br-HTI-TPA-OMe was further encapsulated into nanoparticles using an amphiphilic DSPE-PEG_2000_ and DMPC as the doping matrix to evaluate the feasibility of photocontrolled ^1^O_2_ generation (Figure 2a). As shown in Figure 2b, a bathochromic shift of the maximum absorption peak (NP: *λ*_abs_ = 490 nm vs. hexane: *λ*_abs_ = 480 nm) and a bright fluorescence (*λ*_em_ = 600–640 nm) in water were observed in Z-NPs. In addition, the average hydrodynamic diameter of Z-NPs was determined to be ~122 nm, which is beneficial to efficiently accumulate in the tumor for fluorescence imaging and PDT (Figure 2c). Under 480 nm LED light irradiation, the size of NPs became smaller (~112 nm), resulting in the formation of E-NPs (Figure 2d). As described in Figure 2e, after irradiation with 480 nm, the absorption peak of Z-NPs gradually decreased with a slight red-shifting. The Z-to-E isomerization was nearly saturated within 180 s, indicating that facile photoisomerization could be achieved. Moreover, the initial spectrum of E-NPs fully recovered upon irradiation with the orange-red LED at 580 nm (Figure 2f). After several photoswitch cycles, nanoparticles also displayed good reversibility without obvious attenuation in aqueous solution (Figure 2g). Additionally, upon being irradiated with 480 nm, the emission peak at 640 nm gradually decreased (Figure 2h), indicating that the isomerization process occurred. These results suggested 6Br-HTI-TPA-OMe NPs retained good photochromic performance in the nanostructures, which provides the foundation for in vivo photocontrolled anti-tumor PDT studies. According to DFT calculation, the highest occupied molecular orbital (HOMO) of 6Br-HTI-TPA-OMe (Z isomer) was primarily distributed in TPA moieties due to the strong electron-donating character of N-atom, whereas the lowest unoccupied molecular orbital (LUMO) was mainly located on the thio-part, especially on the carbonyl unit (Figure S9), indicating typical doner-acceptor (D-A) structural features and obvious ICT transition. For 6Br-HTI-TPA-OMe (E isomer), similar HOMO-LUMO orbital distribution was observed.

### 3.3. In Vitro Evaluation of 6Br-HTI-TPA-OMe NPs for Reversible Control ^1^O_2_ Generation

**Figure 3:**
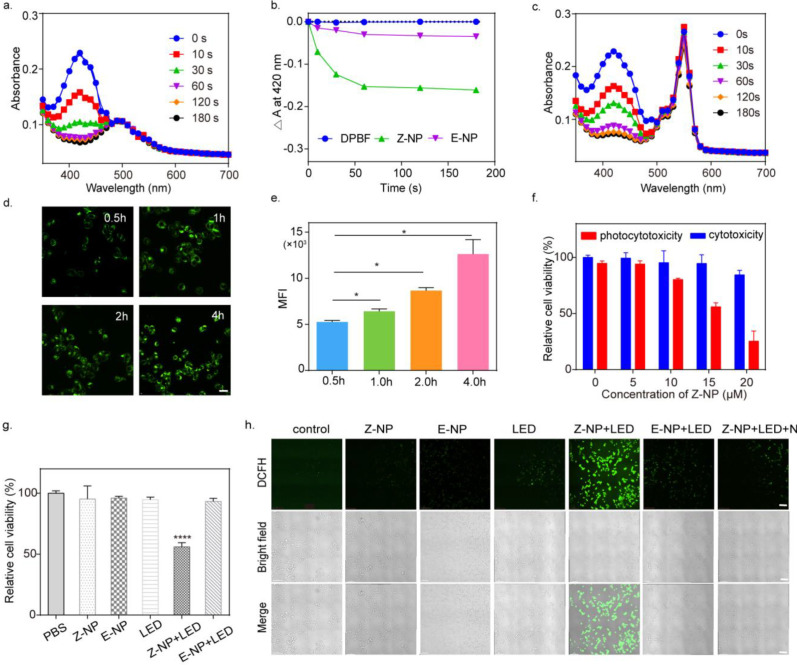
In vitro evaluation of 6Br-HTI-TPA-OMe NPs for reversible control ^1^O_2_ generation. (**a**) Absorption spectrum of DPBF (30 μM) with Z-NP (Z isomer concentration: 10 μM) in water at different irradiations. (**b**) Photodegradation rate of DPBF with or without different nano photosensitizers. (**c**) Absorption spectrum of DPBF (30 μM) with RB (10 μM) in water at different irradiations. Real-time uptake images (**d**) and flow cytometry MFI value (**e**) of Z-NPs (15 μM) in 4T1 cells at different time points (0.5 h, 1 h, 2 h, 4 h). (**f**) MTS assay of Z-NPs at various concentrations co-cultured with 4T1 cells with or without LED irradiation (LED: 520 nm, 40 mW/cm^2^, 5 min). (**g**) MTS assay of 4T1 cells incubated with different treatments. (**h**) Confocal images of 4T1 cells stained with DCFH-DA after different treatments. The treatments included PBS, Z-NPs, E-NPs, LED, Z-NP+LED, E-NP+LED, Z-NP+LED+NaN_3_ (Z isomer concentration: 15 μM, NaN_3_ concentration: 20 mM, 520 nm, 40 mW/cm^2^, 5 min, scale bar: 100 μm). Significant differences between the groups were labelled for * *p* < 0.05, and **** for *p* < 0.0001.

According to the photoswitchable AIE photosensitizer TPE-2Py-DTE [10], the ability to reversible regulation of ^1^O_2_ generation was realized by integrating pyridinium-modified tetraphenylethylene (TPE) photosensitizer moiety and the DTE switch part in one small molecule. Inspired by this, the triarylamine part should function as the AIE photosensitizer building block and double bond in HTI servers as a photoswitch unit in this work, which was expected for realizing photocontrol of the ^1^O_2_ generation. The photocontrolled generation of singlet oxygen in vitro was investigated. Singlet oxygen trapping 1,3-diphenylisobenzofuran (DPBF) was utilized. For reversible ^1^O_2_ generation for Z-NPs and E-NPs, a specific wavelength located at the isosbestic point, a 520 nm LED was selected as the excitation source in order to prevent potential interference. As depicted in Figure 3a, the absorbance of the DPBF at 420 nm dramatically decreased in the presence of Z-NPs upon 520 nm LED irradiation from 0 to 180 s, implying the generation of singlet oxygen. In addition, after isomerization from Z-NPs to E-NPs via exposure to 480 nm, the E-NPs only exhibited a slight decrease in absorption of DPBF under subsequent 520 nm irradiation (Figure 3b). Moreover, the Z-NPs showed a more efficient absorption decay than that of E-NPs, which suggested that Z-NPs are capable of efficiently creating ^1^O_2_ upon irradiation with 520 nm. Furthermore, 6Br-HTI-TPA-OMe NPs showed a comparable ^1^O_2_ generation efficiency compared with commercial Rose Bengal (RB) (Figure 3c and Figure S10). The lowest value Δ*E(_S1−T1)_* of the Z form was calculated to be 0.81 eV, lower than that of the E form (0.84 eV), implying that the oxygen generation via the Z form is easier than the E form in theoretical calculation. Therefore, the theoretical and experimental results supported that reversible Z/E isomerization could photocontrol the ^1^O_2_ generation.

These results implied excellent photo-controllability on ^1^O_2_ production for 6Br-HTI-TPA-OMe NPs. According to these results, the mechanism of 6Br-HTI-TPA-OMe as a photoswitchable AIE PS was proposed. Upon 520 nm LED irradiation, ^1^O_2_ generation was activated in the Z-NPs; after isomerization from Z-NPs to E-NPs, the generation of ^1^O_2_ was suppressed in the E-NPs by the competitive energy transfer. The discrepancies in the triplet energies between the Z isomer and E isomer of HTI may be to blame for this event, resulting in the reversible production of ^1^O_2_ by AIE PS.

Due to the AIE features of 6Br-HTI-TPA-OMe, 4T1 tumor cells were selected for the preliminary evaluation of the cell staining performance. As demonstrated by fluorescence imaging in Figure S11, when 4T1 cells were incubated with Z-NPs, a strong fluorescence signal was visualized in the cytoplasm of 4T1 cells. Moreover, the cellular uptake efficiency of NPs was characterized by using confocal imaging (Figure 3d) and flow cytometry (Figure 3e). It was shown that Z-NPs began to be captured by the cells at 0.5 h and reached a peak at 4 h, suggesting the cellular uptake of Z-NPs was in a time-dependent method.

Encouraged by the photocontrolled ^1^O_2_ generation of 6Br-HTI-TPA-OMe NPs, in vitro PDT therapeutic efficiency was evaluated by MTS assay. After incubating 4T1 cells with different concentrations of Z-NPs (6Br-HTI-TPA-OMe concentration: 0, 5, 10, 15, 20 μM) in the dark (Figure 3f), Z-NPs exhibited negligible dark cytotoxicity with high survival rates, manifesting their excellent biocompatibility. Upon the introduction of 520 nm LED irradiation (40 mW/cm^2^, 5 min), a significant concentration-dependent photocytotoxicity was clearly observed, with the half maximal inhibitory concentration (IC_50_) of 15 μM. Next, 4T1 cells were treated with different nanoparticles either with or without 520 nm irradiation. Here, the group of E-NPs was obtained after 480 nm light irradiation of Z-NPs in cells. As shown in Figure 3g, no obvious toxicity was observed for Z-NPs and E-NPs in dark environments. After irradiation with 520 nm LED (40 mW/cm^2^) for 5 min, the E-NP+LED group exhibited negligible photocytotoxicity (cell viability 93.3%), whereas the survival percentage of 4T1 cells decreased to around 56% in the Z-NP+LED group, showing a significant photocytotoxicity. The results indicated that 6Br-HTI-TPA-OMe NPs could supply accurate PDT treatment by controlling Z/E form via exchanging the irradiation of two lights.

To further confirm the in vitro phototherapeutic mechanism of Z-NPs, the cells were treated with NaN_3_ to scavenge ^1^O_2_ as a control. The generation of intracellular ^1^O_2_ was monitored by using the 2,7-dichlorofluorescein diacetate (DCFH-DA) labeling (Figure 3h). In the group of Z-NPs, E-NPs and LED alone did not trigger the emission of DCFH-DA; the Z-NPs+LED+NaN_3_ group (in which excess NaN_3_ removed ^1^O_2_ completely at first) and E-NP+LED group (in which E-NPs was obtained by isomerization Z-NPs via exposure to 480 nm) only produce weak emission. In contrast, a strong green emission was observed only in the group of Z-NPs+LED. The results suggest that Z-NPs could induce efficient ^1^O_2_ accumulation in cells.

**Figure 4:**
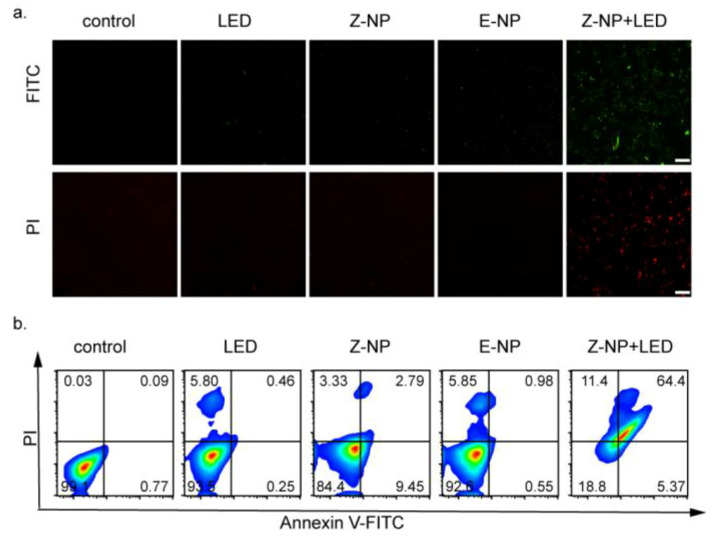
Photodynamic therapy effect of Z-NPs on 4T1 cells assessed by Annexin V-FITC and PI kit. (**a**) Confocal images and (**b**) flow cytometry analysis of apoptosis assay after different treatments. Various therapies were used, including control, LED, Z-NP, E-NP, Z-NP+LED (NP concentration: 15 μM, LED: 520 nm, 40 mW/cm^2^, 5 min, scale bar: 100 μm).

To further identify the different stages of cell apoptosis caused by Z-NPs, stain imaging of 4T1 cells under various treatments was performed using a FITC-Annexin V and propidium iodide (PI) apoptosis kit (Figure 4a). Low levels of cytotoxicity towards 4T1 cells were demonstrated by LED only (520 nm), Z-NPs, and E-NPs, which were consistent with the MTS results. An intense red fluorescence was observed in the Z-NPs+LED group, suggesting that Z-NPs could efficiently kill tumor cells by the generation of ^1^O_2_. These outcomes strongly supported that treatment with Z-NPs and 520 nm illumination renders more 4T1 cells in the apoptotic or necrotic stage. Further investigation about in vivo anti-tumor effects is in process.

## 4. Conclusions

In summary, we have successfully developed a photoswitchable AIE photosensitizer, 6Br-HTI-TPA-OMe, in which TPA moiety and thio-part were covalently linked through an olefinic bond, resulting in efficient ICT between TPA moiety (donor group) and the thio-part (acceptor group), thus permitting reversible control over the production of ^1^O_2_. It showed excellent AIE properties and Z/E photoswitch behavior in various solvents by altering light wavelength (Z to E: 480 nm light; E to Z: 580 nm light). Furthermore, the amphiphilic phospholipid-made nanoparticles (NPs) demonstrated suitable photochromic performance and photocontrolled ^1^O_2_ generation in the aqueous medium. Significantly, the Z-NPs were capable of generating ^1^O_2_ upon 520 nm LED irradiation, but after switching to E-NPs, the generation of ^1^O_2_ was inhibited by the competitive energy transfer, suggesting that reversible Z/E isomerization could photocontrol ^1^O_2_ generation. As anticipated, this photoswitchable AIE photosensitizer performed well in anti-tumor in vitro. The findings in this study could offer a new perspective in the design of functional HTI, and trigger state-of-the-art developments of photoswitchable AIE photosensitizers based on the HTI-TPA skeleton for PDT treatment.

## Data Availability

Not applicable.

## Supplementary Materials

The following supporting information can be downloaded at: https://www.mdpi.com/article/10.3390/bios13030324/s1, Scheme S1: Synthetic route of 6Br-HTI-TPA-OMe; Figures S1–S7: NMR and HRMS spectroscopy; Table S1: UV-vis absorption (*λ_abs_*) and fluorescence (*λ_f_*) maxima, quantum yields of fluorescence (Φ*_f_*), Stokes shifts (Δ*V_st_*) for the Z form of 6Br-HTI-TPA-OMe in different solvents; Figure S8: Absorption and fluorescence spectra in different solvents; Figure S9: The calculated HOMOs and LUMOs; Figure S10: Absorption changes of DPBF with Z-NPs or RB at different irradiations; Figure S11: Confocal images of cellular uptake of Z-NPs by 4T1 cells.
