# Supplementary material for: Rational Design of High-Performance Hemithioindigo-Based Photoswitchable AIE Photosensitizer and Enabling Reversible Control Singlet Oxygen Generation
Source: Biosensors (Basel). 2023 Feb 27;13(3):324. doi: 10.3390/bios13030324 (PMC10046834; doi:10.3390/bios13030324)
Supplement: Supplementary file 1 [file biosensors-13-00324-s001.zip › biosensors-2199037-supplementary.pdf]

Rational design of high-performance hemithioindigo-based photoswitchable AIE photosensitizer and enabling reversible control singlet oxygen generation

Junjie Wang [1], Jianshuang Wei [1], Yuehong Leng [1], Yanfeng Dai [2], Changqiang Xie [2], Zhihong Zhang [1,2], Mingqiang Zhu [2,3,*], and Xingzhou Peng [2,*]

[1] Britton Chance Center and MoE Key Laboratory for Biomedical Photonics, Wuhan National Laboratory for Optoelectronics-Huazhong University of Science and Technology, Wuhan, Hubei 430074, China

[2] Key Laboratory of Biomedical Engineering of Hainan Province, School of Biomedical Engineering, Hainan University, Haikou, Hainan 570228, China

[3] Wuhan National Laboratory for Optoelectronics, School of Optical and Electronic Information, Huazhong University of Science and Technology, Wuhan, Hubei 430074, China

*Correspondence: Xingzhou Peng (pengxzh@hainanu.edu.cn)
Mingqiang Zhu (mqzhu@hust.edu.cn)

Address: Room 205, School of Biomedical Engineering, Hainan University, Haikou, Hainan 570228, China.

Xingzhou Peng (pengxzh@hainanu.edu.cn) is the corresponding author to communicate with the Editorial and Production offices.

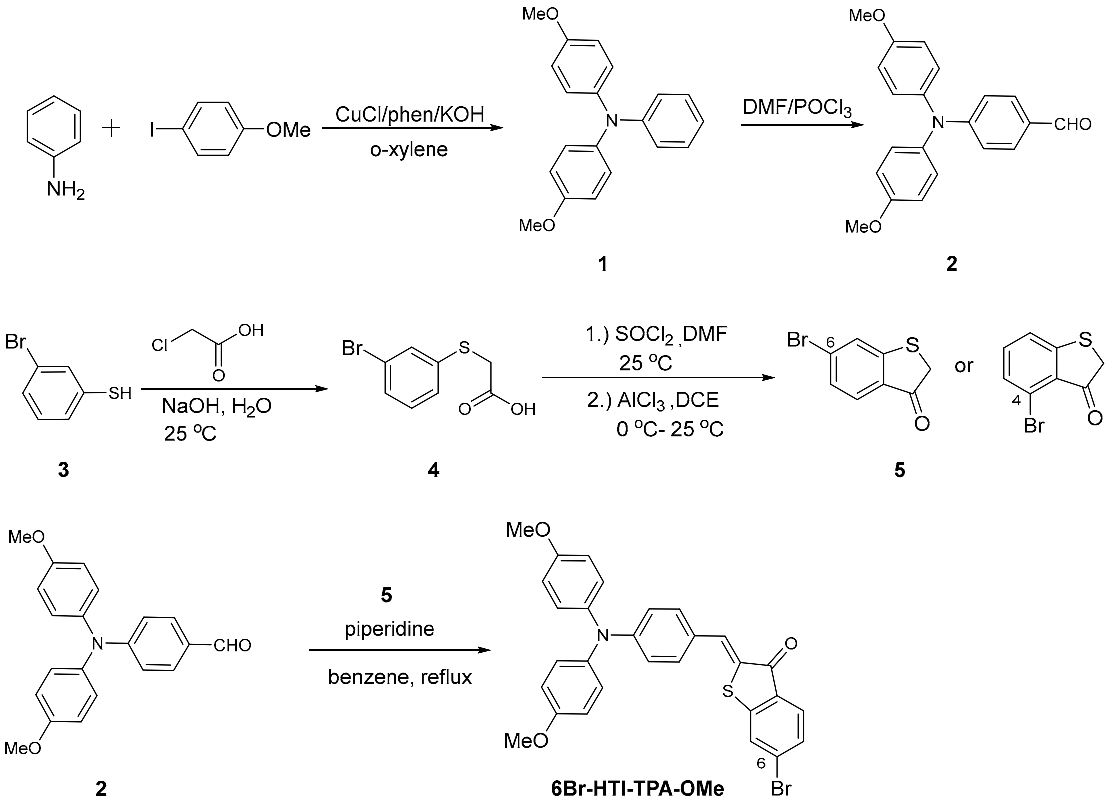

Scheme S1. Synthetic route of 6Br-HTI-TPA-OMe.

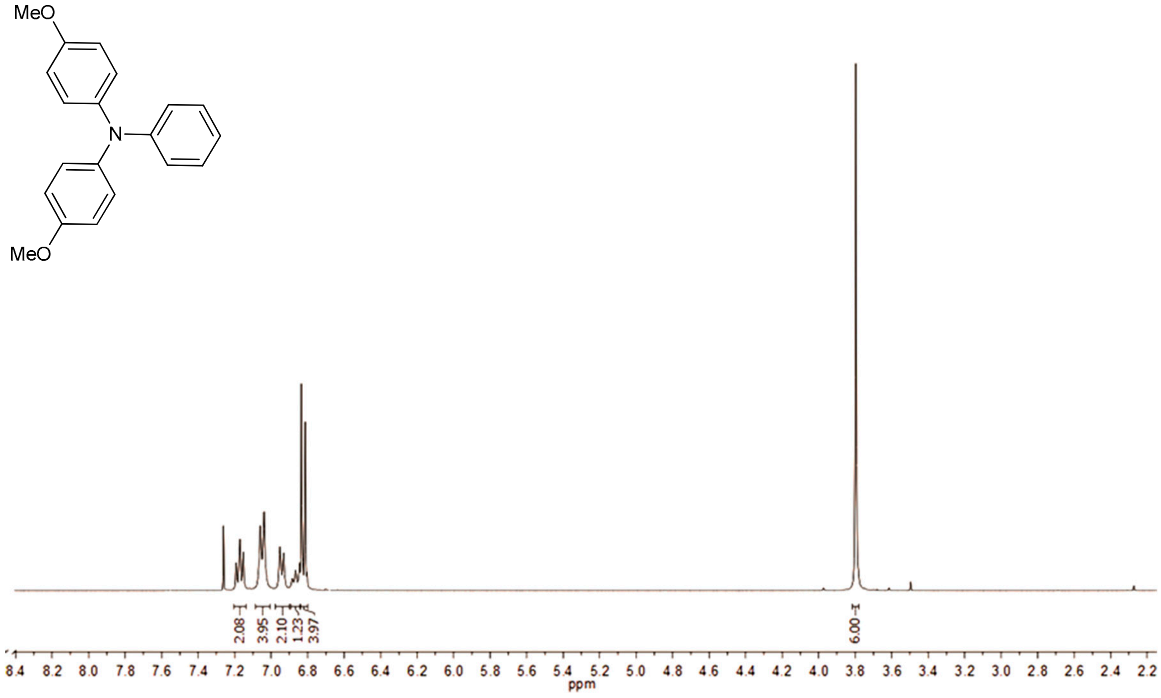

Figure S1. ^{1}H NMR spectra of *N,N'*-di(4-methoxyphenyl)phenylamine **1** in $CDCl_3$ (400 MHz).

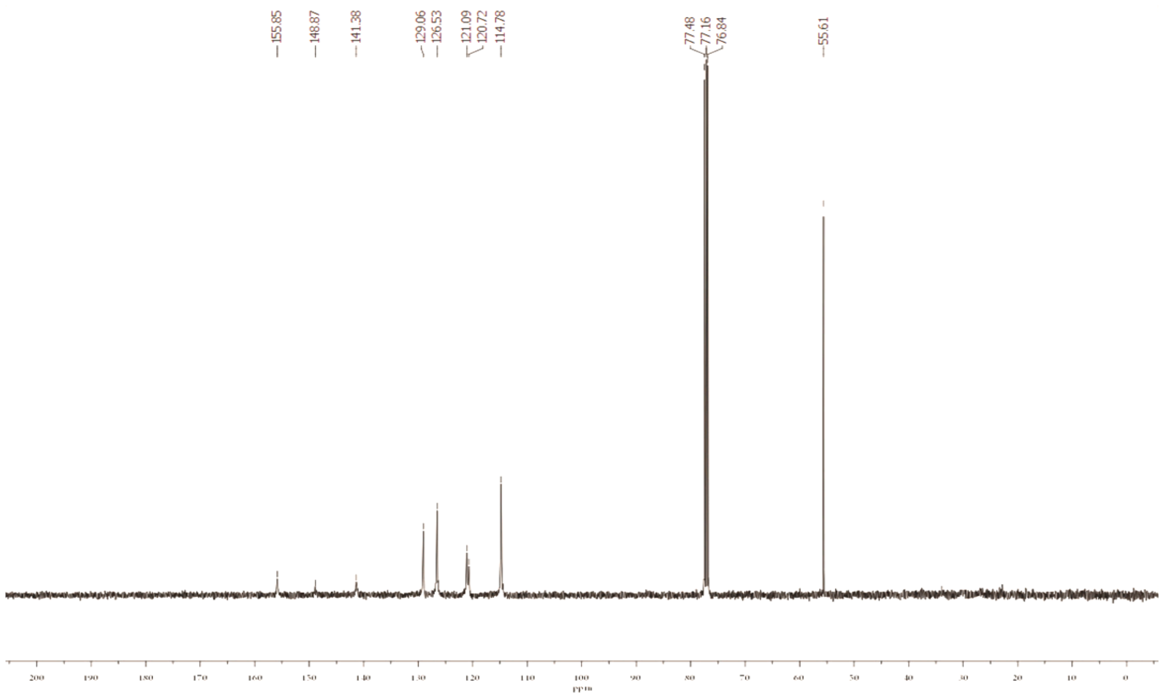

Figure S2.^{13}C NMR spectra of *N,N'*-di(4-methoxyphenyl)phenylamine **1** in $CDCl_3$ (101 MHz).

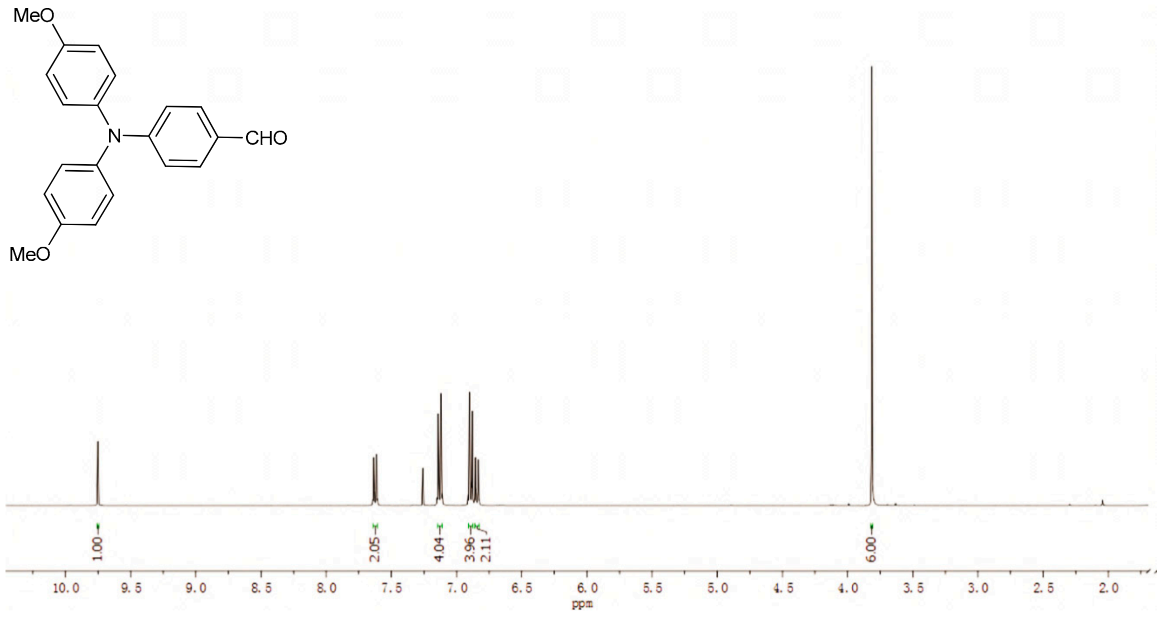

Figure S3. ^{1}H NMR spectra of *N,N'*-bis(4-methoxyphenyl)aminobenzaldehyde **2** in $CDCl_3$ (400 MHz).

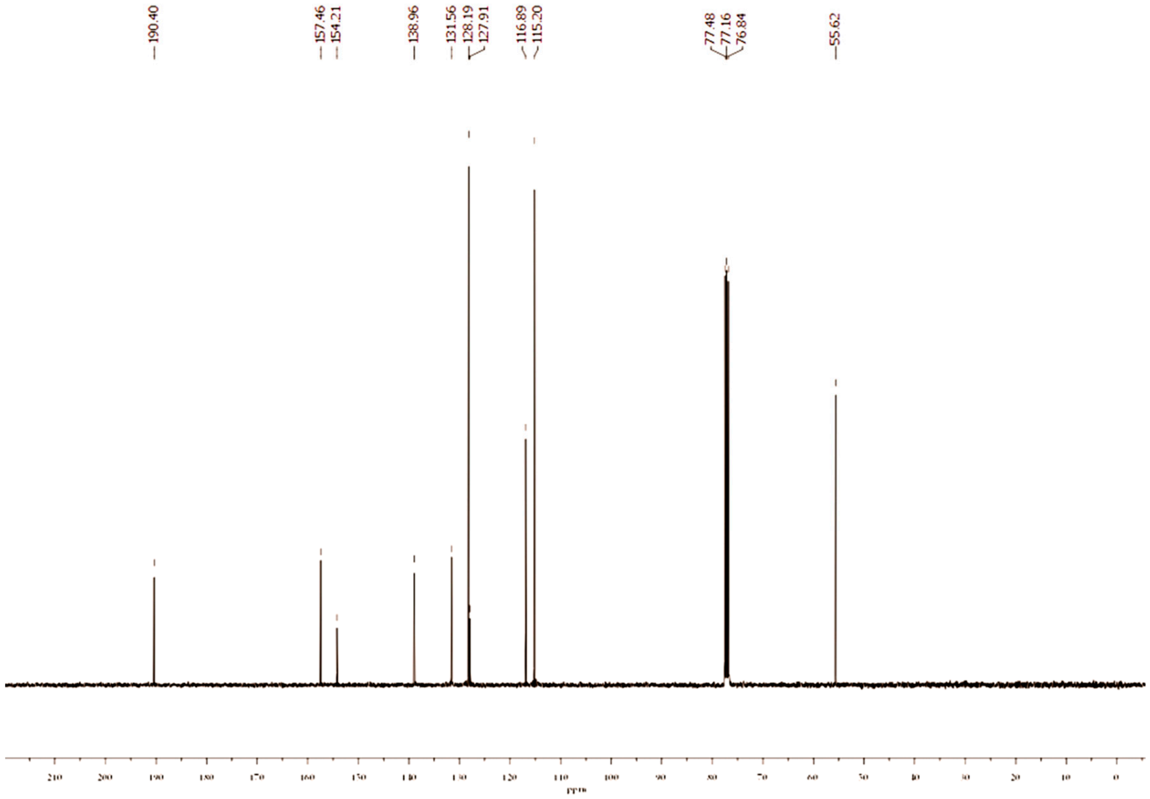

Figure S4. ^{13}C NMR spectra of *N,N'*-bis(4-methoxyphenyl)aminobenzaldehyde **2** in $CDCl_3$ (101 MHz).

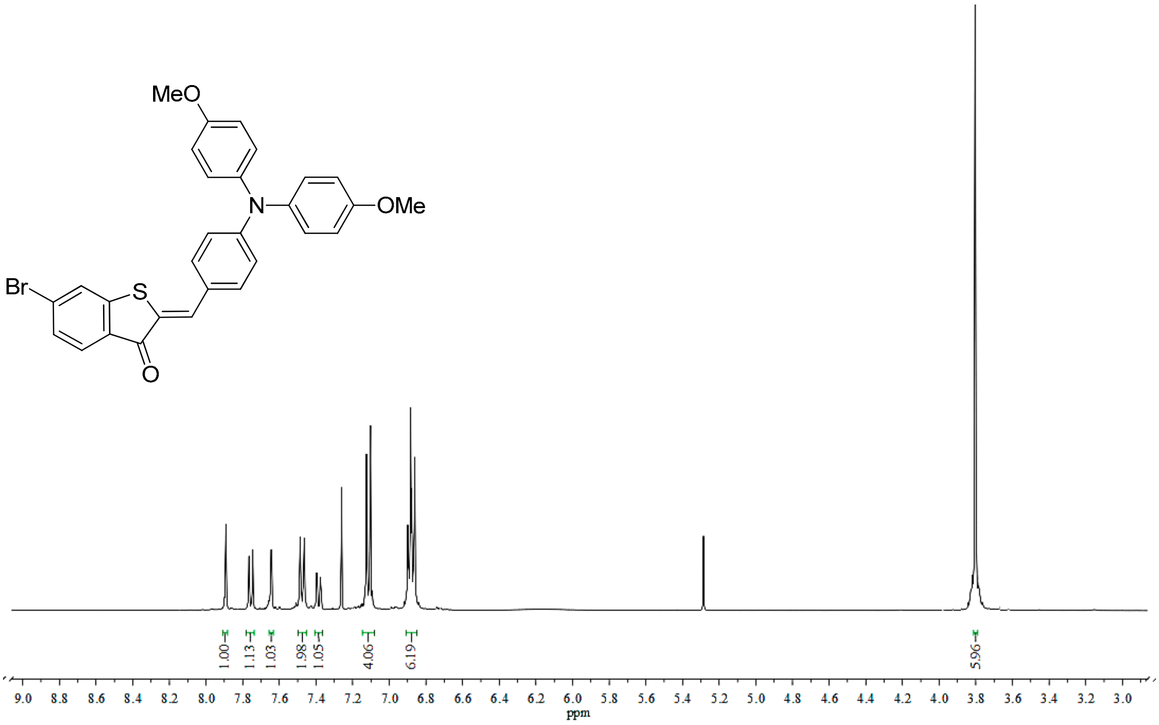

Figure S5. ^{1}H NMR spectra of 6Br-HTI-TPA-OMe in $CDCl_3$ (500 MHz).

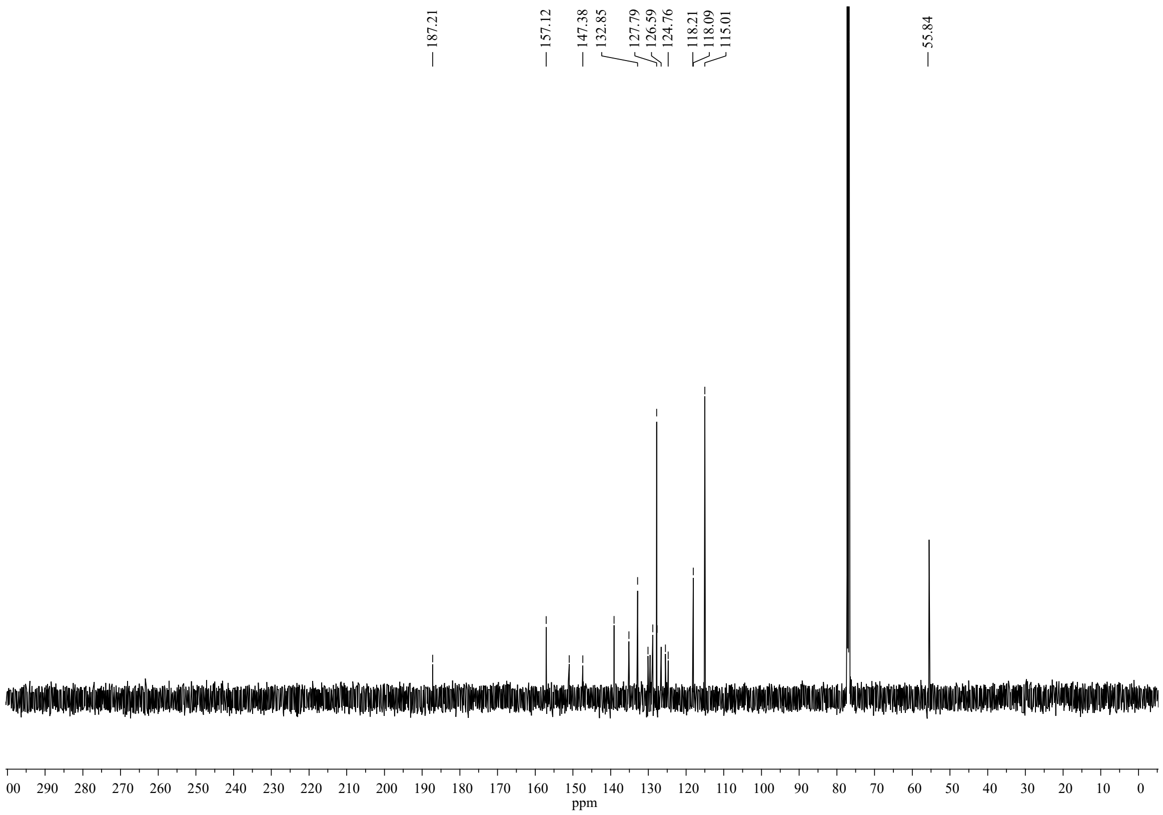

Figure S6.^{13}C NMR spectra of 6Br-HTI-TPA-OMe in $CDCl_3$ (126 MHz).

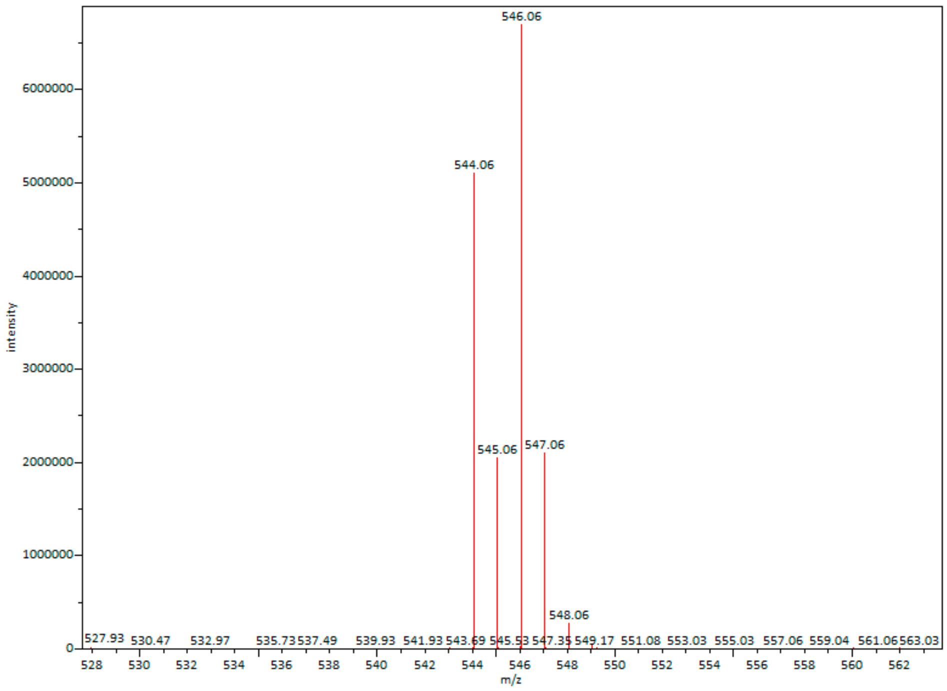

Figure S7. MS spectra of 6Br-HTI-TPA-OMe.

Table S1. UV-vis absorption (λ_{abs}) and fluorescence (λ_f) maxima, quantum yields of fluorescence (Φ_f), Stokes shifts ($\Delta\nu_{st}$) for the Z form of 6Br-HTI-TPA-OMe in different solvents.

solvent	λ_{abs} (nm)	λ_f (nm)	Φ_f (%)	$\Delta\nu_{st}$ (nm)[c]
hexane	488	534	2.8	46
PCE[a]	500	595	-	95
DCM[b]	501	608,713	0.06	107, 212
MeCN	492	621,725	0.01	129, 233

[a] perchloroethylene. [b] dichloromethane. [c] the Stokes shift was calculated from the peak of fluorescence and the absorption maximum.

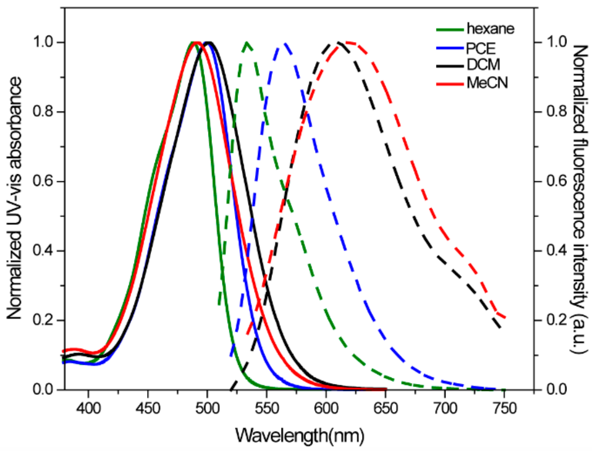

Figure S8. Normalized UV-vis absorption (solid line) and fluorescence (dashed line) spectra of 6Br-HTI-TPA-OMe in different solvents.

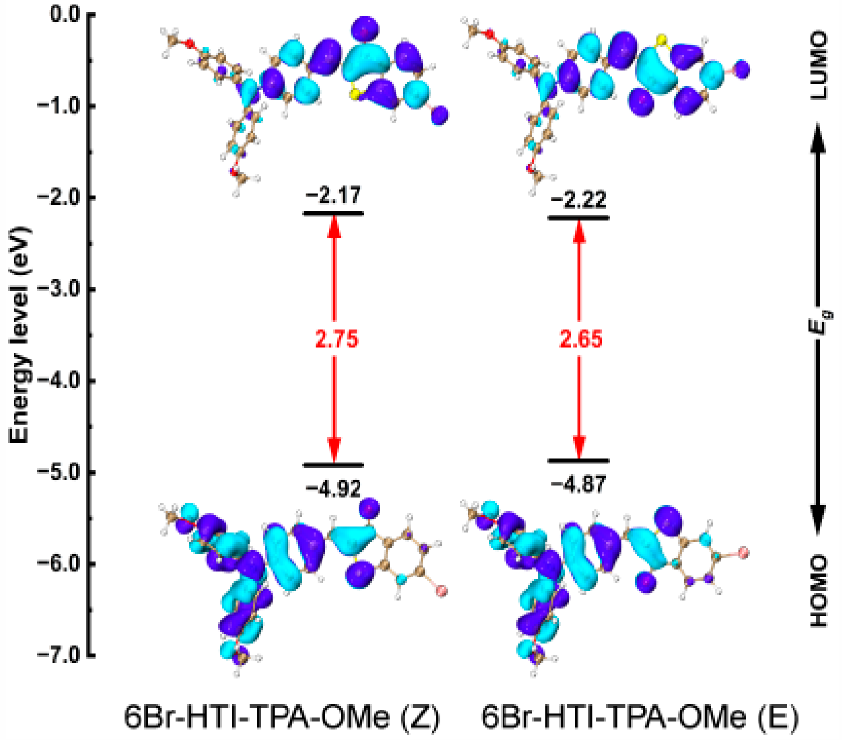

Figure S9. The calculated HOMOs and LUMOs of Z form and E form for 6Br-HTI-TPA-OMe.

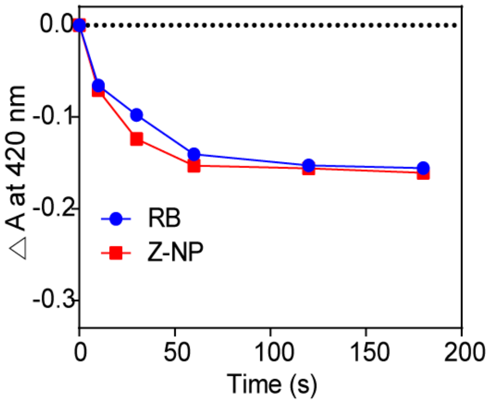

Figure S10. The absorption spectrum of DPBF (30 μM) with Z-NPs (10 μM) or RB (10 μM) in water at different irradiations.

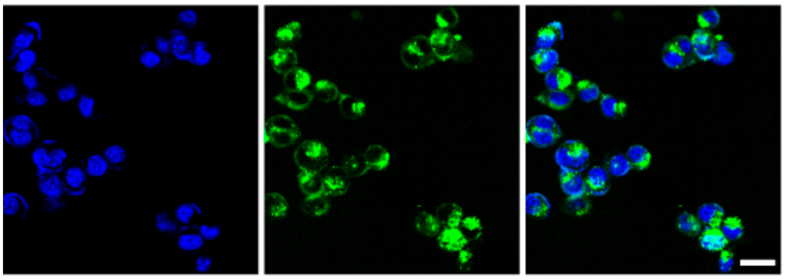

Figure S11. Confocal images of cellular uptake of Z-NPs (5 μM) by 4T1 cells *in vitro* after incubation of 4 h. Blue: nucleus, green: Z-NPs.

EXPERIMENTAL PROCEDURES

Instruments and measurements

Flash-column chromatography was performed using silica gel (60 Å, Sinopharm Chemical Reagent Co. Ltd., China). ^{1}H, ^{13}C NMR spectra were recorded with a Bruker Avance II (400 MHz) in CDCl3 with tetramethylsilane (TMS) as reference at 298 K. Chemical shifts were reported in parts per million (ppm) and were referenced to the residual solvent resonance as the internal standard (CDCl3: δ = 7.26 ppm for 1H NMR and CDCl3: δ = 77.16 ppm for 13C NMR). High-resolution mass spectra (HRMS) were performed on LTQ Orbitrap XL apparatus. All the UV/vis spectra were measured on Shimadzu UV-1601 UV-Visible spectrometer. The fluorescence spectra were obtained

on Hitachi F-4500 fluorescence spectrophotometer. All LED light reached the sample after being focused by a collected lens (5 cm, Thorlabs, COP1-A, Olympus) in the dark room. The light source included 520 nm LED source (3W, 40 mW/cm^2, Shenzhen boya technology), 480 nm LED source (3W, 33 mW/cm^2, Shenzhen boya technology), 580 nm LED source (3W, 54 mW/cm^2, Shenzhen boya technology). In which, the 480 nm LED was used for the Z-to-E isomerization, the 580 nm LED was used for the E-to-Z isomerization. The average hydrodynamic diameter and Zeta potential were measured with Nalvern Nano-ZS90. The data of MTS experiment was measured with Bio-Tek Epoch microplate reader. The confocal imaging experiments were subjected to Andor BC43 (Oxford, USA). The flow cytometry experiments were carried out with Cytoflex S cytometer (Beckman coulter, USA). The ground state (S0) optimized geometry was obtained using B3LYP/6-31G**. The exciton energies of the first singlet (S1) and first triplet states (T1) were obtained on the corresponding optimized ground state structure using TD-B3LYP/6-31G**.

Synthesis of N,N'-di(4-methoxyphenyl)amine

A mixture of 4-methoxyiodobenzene (11.23 g, 48.0 mmol, 2.4 equiv.), aniline (1.86 g, 20.0 mmol, 1.0 equiv.), 1,10-phenanthroline (0.793 g, 4.0 mmol, 0.2 equiv.), CuCl (0.396 g, 4.0 mmol, 0.2 equiv.), KOH (9.28 g, 160.0 mmol, 8.0 equiv.) was dissolved in 50 mL o-xylene and stirred at 160 °C for 12 h. After filtering the solid, the mixture was then extracted with CH_2Cl_2 (25 mL × 3), washed with water (25 mL × 3), the organic layer was decolorized with active carbon for 1 h, then filtered over a pad of Celite and the solvent removed by rotary evaporation to get an oily product. The product **1** was crystallized from methanol to afford the grey crystals (needles, 3.24 g, 53.1 %). ^{1}H NMR (400 MHz, $CDCl_3$) δ = 7.17 (t, ^3J = 7.9 Hz, 2H), 7.05 (d, ^3J = 8.7 Hz, 4H), 6.94 (d, ^3J = 8.0 Hz, 2H), 6.88 (d, ^3J = 7.2 Hz, 1H), 6.85-6.80 (m, 4H), 3.80 (s, 6H); ^{13}C NMR (101 MHz, $CDCl_3$) δ = 155.85, 148.87, 141.38, 129.06, 126.53, 121.09, 120.72, 114.78, 55.61.

Synthesis of N,N'-bis(4-methoxyphenyl)aminobenzaldehyde

To a mixture of *N,N'*-di(4-methoxyphenyl)phenylamine **1** (1.52 g, 5.0 mmol, 1.0 equiv.) and DMF (4.38 g, 60.0 mmol, 12.0 equiv.) at 0 °C, $POCl_3$ (1.53 g, 10.0 mmol, 1.1 equiv.) was added dropwise with stirring. The reaction mixture was stirred at room temperature for 12 h. The reaction was quenched by ice/water and neutralized using NaOH solution (10 mL, 4 mol/L). The mixture was extracted with CH_2Cl_2 (20 mL × 3), washed with water (20 mL × 3), dried over $MgSO_4$ and evaporated under reduced pressure. The purified product **2** was obtained by column chromatography using pentane/ethyl acetate (20:1, v/v) as a yellow powder (0.90 g, 54.2 %).^{1}H NMR (400 MHz, $CDCl_3$) δ = 9.75 (s, 1H), 7.65-7.60 (m, 2H), 7.15-7.11 (m, 4H), 6.92-6.87 (m, 4H), 6.84 (d, ^{3}J = 8.8 Hz, 2H), 3.81 (s, 6H); ^{13}C NMR (101 MHz, $CDCl_3$) δ = 190.40, 157.46, 154.21, 139.96, 131.56, 128.19, 127.91, 116.89, 115.20, 55.62.

Synthesis of 2-((3-bromophenyl)thiol)acetic acid

3-Bromobenzenethiol **3** (3.78 g, 20 mmol, 1.0 equiv.) was dissolved in NaOH solution (1.60 g in 20 mL H_2O) under N_2 atmosphere. The solution of chloroacetic acid (2.07 g, 22 mmol, 1.1 equiv.) was added drop-wise to the mixture at 0 °C. The reaction mixture was stirred at room temperature for 18 h. After complete consumption of the starting material (TLC monitoring), the mixture was acidified with HCl solution (25 mL, 6 mol/L) and precipitated. The crude product was washed with water and dried over P_2O_5, yielding 2-((3-bromophenyl)thiol)acetic acid **4** (white solid, 4.92 g, 92.8 %). m.p. 118.0 °C (reference reported 117 - 119 °C).

Synthesis of 4-/6-bromobenzo[b]thiophen-3(2H)-one

2-((3-Bromophenyl)thiol)acetic acid **4** (2.46 g, 10.0 mmol, 1.0 equiv.) was dissolved in $SOCl_2$ (27.9 g, 230.0 mmol, 23 equiv.) and a few drops of DMF were added under N_2. Then the reaction mixture was stirred at room temperature for 2 h. The excess of $SOCl_2$ was removed by distillation under reduced pressure. The resulting crude acid chloride was dissolved in 20 mL DCE and the solution was cooled to 0 °C. Aluminum chloride (1.7 g, 13.0 mmol, 1.3 equiv.) was added in 2-3 portions over 5 min at 0 °C. The mixture was stirred under N_2 at 25 °C for 80 min. The reaction was quenched with ice/water

and extracted with CH_2Cl_2 (20 mL × 3). The organic solution was washed with saturated sodium chloride (20 mL × 3), dried over anhydrous $MgSO_4$, filtrated and evaporated to give the crude product, a mixture of 4-/6-bromobenzo[*b*]thiophen-3(2*H*)-one **5** (4Br : 6Br = 27 : 73). This crude product was used for the next step directly (1.49 g, 65.4 %).

Encapsulation efficiency

The 6Br-HTI-TPA-OMe NPs were purified via high-speed centrifugation to remove residual 6Br-HTI-TPA-OMe small molecules in the supernatant. The amount of 6Br-HTI-TPA-OMe was determined as follows:

$$\text{Encapsulation efficiency (\%)} = W_t/W_i \times 100\%$$

Where W_t is the actual amount of the 6Br-HTI-TPA-OMe in the formulation, which was detected by UV-vis spectroscopy at 520 nm, and W_i is the amount of the initial amount of the 6Br-HTI-TPA-OMe added during the preparation process.

Fluorescence quantum yield

The quantum yields of fluorescence (Φ_f) were obtained by a reference method with RhB (Φ_f = 0.7, methanol) as the standard compound. The calculation of the quantum yield of the samples was based on equation (1) as followed:

$$\emptyset_f = \emptyset_{f(std)} \frac{F \cdot A_{std} \cdot n^2}{F_{std} \cdot A \cdot n_{std}^2}$$

Where F and F_{std} are the integrated fluorescence area of samples and standard; A and A_{std} are the absorbance of samples and standard; n and n_{std} are the refractive index of H_2O and methanol, respectively.

$^1O_2$ generation

The singlet oxygen generation efficiency was to test in aqueous solution. 1,3-diphenylisobenzofuran (DPBF) was first dissolved in DMSO to give a 20 mM of solution, and then diluted with H_2O to 30 μM (containing 0.15% DMSO). The DPBF solution (200 μL) was then mixed with Z-NPs or E-NPs (containing [DPBF] = 30 μM,

[NPs] = 10 μM). The mixture was irradiated with 520 nm LED light immediately, and then DPBF degradation at 420 nm was monitored along with irradiated time.

Cell uptake experiment

The uptake of 6Br-HTI-TPA-OMe NPs in the Z form by tumor cells was studied in 4T1 cells. The Z-NPs (5 μM) and cells were co-incubated for 0.5h, 1h, 2h and 4h, respectively. The real-time imaging was performed to monitor cell uptake using an an LSM 710 laser confocal scanning microscope (Zeiss, Germany, Ex: 488 nm, collection channel: 500-700 nm)

***In vitro* cytotoxicity assays**

For the *in vitro* photodynamic therapeutic effect and cytotoxicity of Z-NPs, the MTS assay was used to analyse the viability of 4T1 cells. Cells were seeded in 96-well plates (1×10^4 cells per cell) and incubated for 24 h at 37 °C under 5% CO_2. Subsequently, the cells were incubated with different concentrations (0, 5, 10, 15 and 20 μM) of Z-NPs for 4 h and irradiated with a 520 nm LED light (40 mW/cm^2) for 5 min or without light. After washing with PBS three times, they were incubated at 37 °C for another 20 h. Then, 100 μL of 10% [3-(4,5-dimethylthiazol-2-yl)-5-(3-carboxymethoxyphenyl)-2-(4-sulfophenyl)-2H-tetrazolium] (MTS) was added to each well and cultured for 2 h at 37 °C. The absorbance at 490 nm was then measured using a microplate spectrophotometer to calculate the cell survival rate.

Intracellular $^1O_2$ detection

The $^1O_2$ indicator DCFH-DA was used to detected $^1O_2$ produced Z-NPs after irradiating with 520 nm LED light in 4T1 cells. In brief, 4T1 cells was incubated with different subsequent treatments: (1) control; (2) Z-NPs, incubated with 15 μM Z-NPs for 6 h, then washed three times with PBS, incubated with 30 μM DCFH-DA for 20 min; (3) LED, incubated with 30 μM DCFH-DA for 20 min, then washed three times with PBS, and irradiated with 520 nm LED light for 5 min; (4) Z-NP+LED, incubated with 15 μM Z-NPs for 6 h, then washed three times with PBS, incubated with 30 μM DCFH-DA for 20 min, then washed three times with PBS, and irradiated with 520 nm LED light

for 5 min; (5) Z-NP+LED+NaN_3, pre-incubated with 20 mM NaN_3 for 1 h, then incubated with 15 μM Z-NPs for 6 h, then washed three times with PBS, incubated with 30 μM DCFH-DA for 20 min, then washed three times with PBS, and irradiated with 520 nm LED light for 5 min. After these treatments, confocal fluorescence imaging was performed to detect the level of singlet oxygen (Ex: 488 nm, collection channel: 510-600 nm).

Apoptosis test

FITC-Annexin V/propidium iodide (PI) Apoptosis Detection Kit (Shanghai Yesen Biotechnology Co. Ltd) was used to perform double-stain imaging of photo-induced cell death of 4T1 cells. In brief, 4T1 cells were seeded in confocal dishes and then incubated with Z-NPs (15 μM), E-NPs (irradiated with 480 nm LED to form E-NPs, 15 μM) for 4 h, then irradiated with or without 520 nm LED light (40 mW/cm^2) for 5 min. After treatment, the cells were stained with a FITC Annexin V/PI Apoptosis Detection Kit according to the manual. The apoptosis imaging was observed by confocal microscope imaging (Ex: 488 nm for FITC, Ex: 535 nm for PI). In addition, the fluorescence intensity of the above treated cells was quantitatively analyzed by flow cytometry.

Statistical analysis

Statistical analysis was performed using GraphPad Prism (GraphPad Software, Inc., La Jolla, CA, USA). For comparisons of two groups, a two-tailed unpaired *t*-test was performed. All statistical data are expressed as the mean ± SD. Significant differences between the groups were labelled * for $p < 0.05$, ** for $p < 0.01$, *** for $p < 0.001$ and **** for $p < 0.0001$.
